# Widowhood and Health Status Among Chinese Older Adults: The Mediation Effects of Different Types of Support

**DOI:** 10.3389/fpubh.2021.745073

**Published:** 2021-11-17

**Authors:** Yu Guo, Tingshuai Ge, Li Mei, Lina Wang, Jingbo Li

**Affiliations:** ^1^School of Public Policy and Administration, Institute for Population and Development Studies, Xi'an Jiaotong University, Xi'an, China; ^2^School of Labor Economics, Capital University of Economics and Business, Beijing, China

**Keywords:** widowhood, social support, health, mediation effects, older adults

## Abstract

Although many studies have suggested that widowhood is related to worse health conditions among older adults, few have examined the mediation effects of social support between widowhood and health. Employing mediation analysis to a sample of data from the 2014 wave of China Longitudinal Aging Social Survey (CLASS), this study examined the mediation effects of social support, including emotional support, instrumental support, and companionship, in the widowhood-health association among older adults. The results indicated that the negative effect of widowhood on older adults' health was in part attributable to decreased emotional support and companionship. Specifically, emotional support exerted a significant role in the widowhood-mental health association, and companionship exerted a significant role in widowhood-physical health and widowhood-mental health associations. In the subsample analysis, the mediation effects were only significant among female older adults, and among rural older adults. Our findings highlight the importance of emotional support and companionship in maintaining health among widowed older adults and strategies should pay more attention to female and rural widowed older adults.

## Introduction

The loss of a spouse is one of the most painful and stressful events faced by older adults, as well as an inevitable role transition (1–3). Surviving spouses must bear the grief of losing a partner who provides daily support and companionship (4, 5). This may have adverse effects on mental and physical health (6–9). Even if widowhood is universal for all older adults, its impact on health varies according to gender, cultural norms, and social structure (10, 11).

A recent study indicates that widowhood alone may not lead to depression in older adults, but those who are widowed and socially isolated have an increased risk of depression (11). Social support seems to be a potential mediator in the pathway from widowhood to health. Lots of research has proved that social support as an important protective factor for mental and physical health, can lower the mortality risk, prevent depression, and maintain good physical health among older adults. However, social support tends to shrink with age, particularly after the death of a spouse who may be the primary support provider (12, 13). Currently, we know little about the role of social support and its subtypes in the relationship between widowhood and health. Besides, considering the characteristics of social support formed by different social structures and cultural norms are different (11, 12), it is necessary to explore the role of support in the pathway from widowhood to health, under the background of urban-rural dual structure and rigid gender division.

Therefore, using the 2014 wave of China Longitudinal Aging Social Survey (CLASS), this paper aimed to examine: (1) whether the relationship between widowhood and the health status of older adults is mediated by social support, especially the different types of social support; (2) whether the mediation effects of social support vary according to gender and rural-urban areas. Below, we first introduced the literature review, which provides an overview of the relationship between widowhood, social support, and health status, and a brief introduction of the Chinese context. Second, the data and methods section outlines the sample adopted, the measurement of variables, and the analytic strategy. Third, the results section demonstrates the role of social support in the widowhood-health relationship, including gender and rural-urban specific findings. Finally, the discussion and conclusions section compares the findings to the existing literature and considers the contribution to the existing knowledge base. And the limitations of the study and suggestions for further research are also considered.

## Literature Review

### Widowhood and Health Status

Many studies have shown that the health of older adults tends to deteriorate after losing a spouse (14–16). According to the “marriage protection effect” hypothesis, marriage can promote good health through support for healthy behaviors (17). For example, spouses can motivate or encourage each other to quit smoking, engage in physical activities, and maintain a healthy diet, all of which are important for somatic health (18). On the other hand, marriage can also promote good health due to the available companionship, economic, and material resources (19, 20). A study by Emanuele reported that co-residency of couples provides companionship, better financial status, and health care resources (21).

The association and the mechanisms between widowhood and health seem not the same for all age groups (5, 10, 22). It is more detrimental to be bereaved at a younger age than it is to be widowed later in life (10). Widowhood, for example, raises the risk of death in older people, but the link is weaker for those aged 75 and above (22). Based on a sample of 75 years old and blow, Golden et al. (23) found that a higher prevalence of depression in widows with a higher prevalence of loneliness and isolation. While Forster et al. (24) found that widowhood alone is not necessarily related, only the oldest widowers, those socially isolated, face an increased risk of depressive symptoms.

Further studies also suggest this protection effect differs by gender. Nonetheless, the findings are mixed. For example, several studies hold that men are perceived to gain more health benefits from marriage than women (6, 25), and thus men are always more vulnerable during the transition to widowhood (26, 27). While some other scholars believe that in the context of patriarchy, such as in China, women gain more health benefits from marriage due to gender inequality (11, 28). Because women tend to experience disadvantages in labor markets and face a greater risk of poverty than men, staying in marriage allows women to enjoy better living conditions and health benefits. Thus, women are more negatively affected and are more likely to suffer from poor health after being widowed (29).

### Widowhood and Social Support

An abundance of research has shown that widowhood is linked to a decrease in social support (23, 30–32). Widowhood means the loss of primary support from the deceased spouse. More importantly, with the death of a spouse, relatives, and friends who relied on the spouse to maintain close relationships gradually move away, and the support from their relatives and social networks gradually decline (5, 33), resulting in less social support for the older adults (5). Furthermore, widowhood also leads to poor health status among older adults, creates barriers to social participation and maintaining social connections, and results in changes in social networks and less social support (34). Although some research focused on marital status, and found that widowers received less support than their coupled counterparts (14, 29), little has differentiated the types of social support, such as emotional and instrumental support (29).

Further research suggests that the change in social support for widowed older adults differs by gender. That is, for husbands, widowhood means the loss of the wives' companionship and daily care. And for wives, widowhood means a decline in financial and practical support more than in companionship and daily care. Meanwhile, in marriage, husbands tend to benefit from their wives' network of social support, whereby men might experience difficulty in maintaining relationships with relatives and friends after becoming widowed (35–37).

### Social Support and Health

Social support refers to the actual or perceived availability of resources from the individual's social networks (38), and it has been proven to have a significant positive association with health throughout one's lifespan (39–41). Social support can be classified into emotional support (e.g., providing empathetic understanding and warm care, talking about problems), instrumental support (e.g., entailing help with household tasks or financial aid through goods or services), and companionship (e.g., daily meetings and contact) (42–44). Instrumental support refers to tangible help, such as providing goods or services. Emotional support implies psychological help, entails talking about problems, privacy, and providing advice, and companionship focused on the accompanying of a variety of daily activities (43, 44).

Different types of social support may exert different protective effects on health. Most researchers are interested in emotional and instrumental support and hold it strongly associated with overall health and well-being (42), and it can reduce the risk of dementia and mortality (45, 46), help older adults stay healthy and relieve their psychological stress and burden (13, 47). There are fewer concerns about companionship support, however, they are often supportive in their own right (48, 49). Some researchers focus on the influence of social contacts on health and show significant and meaningful associations between contact frequency and health outcomes (50). Companionship means daily meeting and spending time together, which further influences health status by affecting the mood, attitude, and cognition of widowed older adults (51).

Men and women may receive different types and levels of social support, which may result in different outcomes in health (52). For example, a study found that men benefit from social support in terms of improved mental health, while women benefit from social support in terms of improved physical health (53). Another study on marital loss discovered that perceived social support has a moderate and significant effect on marital transition in men but not in women (29).

### Chinese Context

China has a large population of widowed older adults. The number of widowed older adults in China has continued to increase since the country transitioned into an aging society in 2000. According to recent survey data, 27.9% of the population aged 65 and older in China are widowed (16.0% for men and 38.6% for women), accounting for a large share of the global population of widowed older adults (2, 54).

Family orientation and gender division are rooted in Chinese society and culture. Family members, especially spouses, are always the primary provider of support. This situation is particularly typical in rural China, where it is characterized as “hollowing,” that is, young people go out to work, leaving the elderly parents to live alone and support each other. Therefore, widowhood may deal a heavier blow to the elderly in China than in western countries. Besides, the Chinese culture is characterized by patrilocality, patrilineality, and patriarchy, in which wives depend heavily on their husbands (55). In such a context, the death of the spouse, who is the most essential and longest-standing family member with older adults, can generate an even more adverse effect on the health of China, and this impact may have gender differences.

Moreover, there is a long-standing urban-rural dual system in China which has in some ways created a marked difference in social networks (56, 57) and medical resources (58, 59). This too could pose a mechanism affecting the health status of widowed older adults. Besides, the social security system in China is less developed and inadequate to provide comprehensive protection for widowed older adults (60), especially in rural areas.

### The Present Study

Based on the overview of previous studies, although many studies have examined the relationship between widowhood and health, few have explored whether social support, as well as subtypes, play a mediating role in the relationship between widowhood and health, and the potential gender and urban-rural differences. Our study aims to examine the role of social support in the widowhood-health association of older adults and provide gender and rural-urban-specific analysis. Understanding this can inform policymakers to take more effective measures to help bereaved older adults adjust to life after widowhood and improve their health, which is essential for healthy aging.

## Data and Methods

### Data

The data used in this study was from the China Longitudinal Aging Social Survey of 2014 (CLASS). CLASS is a nationwide, continuous, large-scale social survey project conducted by the Renmin University of China in 2014. Applying the multi-stage stratified probability sampling method and face-to-face interview, CLASS covered 462 villages or communities across 29 provinces, a total of 11,511 participants aged 60 and above in China (excluding Hong Kong, Taiwan, Macau, Hainan, Xinjiang, and Tibet). The data collection was approved by the Ethics Committee of Renmin University of China, and each participant was informed of the purpose of this survey. The participation of each participant in the study was voluntary, and they were assured that their privacy would be strictly protected. Given the purpose of this study that examined the mediating role of social support between widowhood and older adults' health status, all participants who had never been married or divorced were excluded. Additionally, we excluded participants who did not complete the mental health questionnaire due to cognitive impairment (61, 62). Finally, participants with missing values in the variables of interest were excluded. The final analysis sample includes 7,647 participants, among whom 28% were widowed, 46% were women, and 45% lived in rural areas.

### Variables

#### Health Status

Health status, which usually includes mental health and physical health, is a crucial indicator of successful aging (63, 64). For mental health, it was measured using the abbreviated 12-item Center for Epidemiologic Studies Depression scale (CES-D) (65). This instrument is widely used to measure the mental health of older and widowed adults (66–68). The scale includes three questions on positive affect (feeling happy, enjoying life, feeling pleasure), two questions on negative affect (feeling lonely, upset), five questions on marginalization (feeling useless, having nothing to do, unaccompanied, isolated, and neglected) and two questions on somatic symptoms (poor appetite, trouble sleeping). Each item was scored on a scale (0 = most time; 1 = sometimes; 2 = hardly ever), suggesting the frequency of the symptom they experienced last week (the positive items were reverse coded). The sum score ranged from 0 to 24, with higher scores indicating better mental health (Cronbach's α = 0.756).

For physical health, it was assessed by using 10 basic items of the Activities of Daily Living scale (ADL): cleaning, dressing, bathing, self-feeding, controlling bowel and urine, toileting, transferring from bed to chair, indoor transferring, and climbing stairs. Each item was rated on a 3-point scale based on the individual's ability to perform the activities (0 = unable; 1 = with help; 2 = on my own). Higher final sum scores (range = 0–20) are associated with better physical health (Cronbach's α = 0.887).

#### Widowhood Status

According to the purpose of the study, we focused on older adults whose marital status is widowed or married with a spouse (non-widowed). Widowhood status was measured by asking the participants' current marital status, and widowhood status was coded as 1 if widowed; otherwise, coded as 0.

#### Social Support

Social support was measured using the Lubben Social Network Scale (LSNS), a 6-item scale assessing the availability of social support from friends, relatives, and neighbors. The scale estimates the number of people to talk about privacy, help in need, and contact or meet, using items such as “How many families/relatives do you feel comfortable talking about private matters with?” “How many families/relatives are available to you when you need them?”, and “How many families/relatives do you see or contact at least once a month.” And then the same set of questions were prompted in regards to their relationship with friends. According to the previous research using CLASS data and the scores assigned by the CLASS questionnaire: none (0), one person (1), two persons (2), three to four persons (3), five to eight persons (5), and nine persons and above (9). We added up the scores for each question for each type of support and created a variable whose values ranged from 0 to 54 (69) (Cronbach's α = 0.778). Further, we use the total number of friends, relatives, and neighbors who can talk, help and meet to measure the emotional, instrumental and companionship support. Each subtype of social support ranges from 0 to 18. A higher value indicates stronger perceived social support.

#### Covariates

Many covariates were controlled in this study based on prior research (70, 71), including age (60–74 = 0, 75 and above = 1), gender (male = 0, female = 1), ethnicity (Han = 0, minority = 1), hukou type (agricultural = 0, non-agricultural = 1), education level (primary and below = 0, junior high = 1, secondary = 2, university and above = 3), whether with religion (no = 0, yes = 1), participation in community activities (policing patrols, caring for other older adults, environmental protection, mediating disputes, etc.) (no = 0, yes = 1) and living with someone (no = 0, yes = 1).

### Analytic Strategy

We used SPSS Macro PROCESS to test the mediation effect of social support in the relationship between widowhood and health among older adults. PROCESS adopts a bias-corrected percentile bootstrap method which is one of the most valid and robust methods for testing mediation effects. Results obtained using this procedure have a higher likelihood of being devoid of Type I error and also estimated more accurate confidence intervals (72–74). An indirect effect is considered significant when the confidence interval does not include a zero.

Below we first described the characteristics of the variable used in this study by adults' widowhood status. Mediation analysis was conducted to examine the mediation effect of social support between widowhood and older adults' health status in the total, male-female subsample, and rural-urban subsample. We conducted a causal mediation analysis to test the reliability of the results. The mediation effect of social support calculates using a sequential approach proposed by Hick and Tingley (75). Following Robins and Greenland (76) and Pearl (77), this method calculates the average of the mediation effects between the actual outcome and counterfactual outcomes to estimate the indirect effects. This causal mediation analysis was performed using STATA 15.1.

## Results

### Descriptive Results

Descriptive and chi-square test results between widowed and non-widowed participants are shown in Table 1. Numerical variables were reported as mean ± standard deviation (SD). Categorical variables were reported as proportions (%). As shown in Table 1, there are significant differences in health status and social support between non-widowed and widowed older adults. Specifically, widowed older adults have poorer mental and physical health status, and receive less emotional, instrumental, and companionship support.

**Table 1:** Descriptive statistics for widowed vs. non-widowed participants (*N* = 7,647).

**Variables**	**Widowhood status**		***P*** **-value**
	**Non-widowed** **(***N*** = 5,518)**	**Widowed** **(***N*** = 2,129)**	
Mental health	12.66 ± 2.24	11.93 ± 2.76	<0.001
Physical health	19.70 ± 1.35	19.42 ± 1.80	<0.001
Social support	19.85 ± 11.39	18.91 ± 10.77	<0.010
Emotional support	5.06 ± 4.10	4.80 ± 3.75	<0.010
Instrumental support	6.51 ± 4.42	6.36 ± 4.25	0.175
Companionship	8.28 ± 4.59	7.76 ± 4.43	<0.001
Gender (%)			<0.001
Male	61.44	32.60	
Female	38.56	67.40	
Age (%)			<0.001
60–74	85.85	58.48	
≥75	14.15	41.52	
Ethnicity (%)			0.047
Han	93.71	93.24	
Ethnic Minority	6.29	6.76	
Hukou type (%)			<0.001
Agricultural	43.64	48.90	
Non-agricultural	56.36	51.10	
Education level (%)			<0.001
Below elementary school	51.54	70.88	
Junior high school	26.21	15.97	
High school	13.56	8.97	
College and above	8.70	4.18	
Religion (%)			<0.001
No	90.16	84.78	
Yes	9.84	15.22	
Social participation (%)			0.023
No	70.66	73.27	
Yes	29.34	26.73	
Live with someone (%)			0.226
No	0.05	0.14	
Yes	99.95	99.86	

*Numerical variables were reported as mean ± standard deviation (SD); Categorical variables were reported as proportions (%); P-value denotes statistical significance for comparison between non-widowed and widowed old adults, based on chi-square or t-statistics test*.

### Mediation Analysis

We further follow the procedure of mediation effects analysis proposed by Zhao et al. (72) and refer to the bootstrap test for mediation effects proposed by Preacher and Hayes (73, 78), with 5,000 bootstrap samples to obtain the bias-corrected 95% confidence intervals for the total indirect effect and the specific indirect effects.

**Figure 1:**
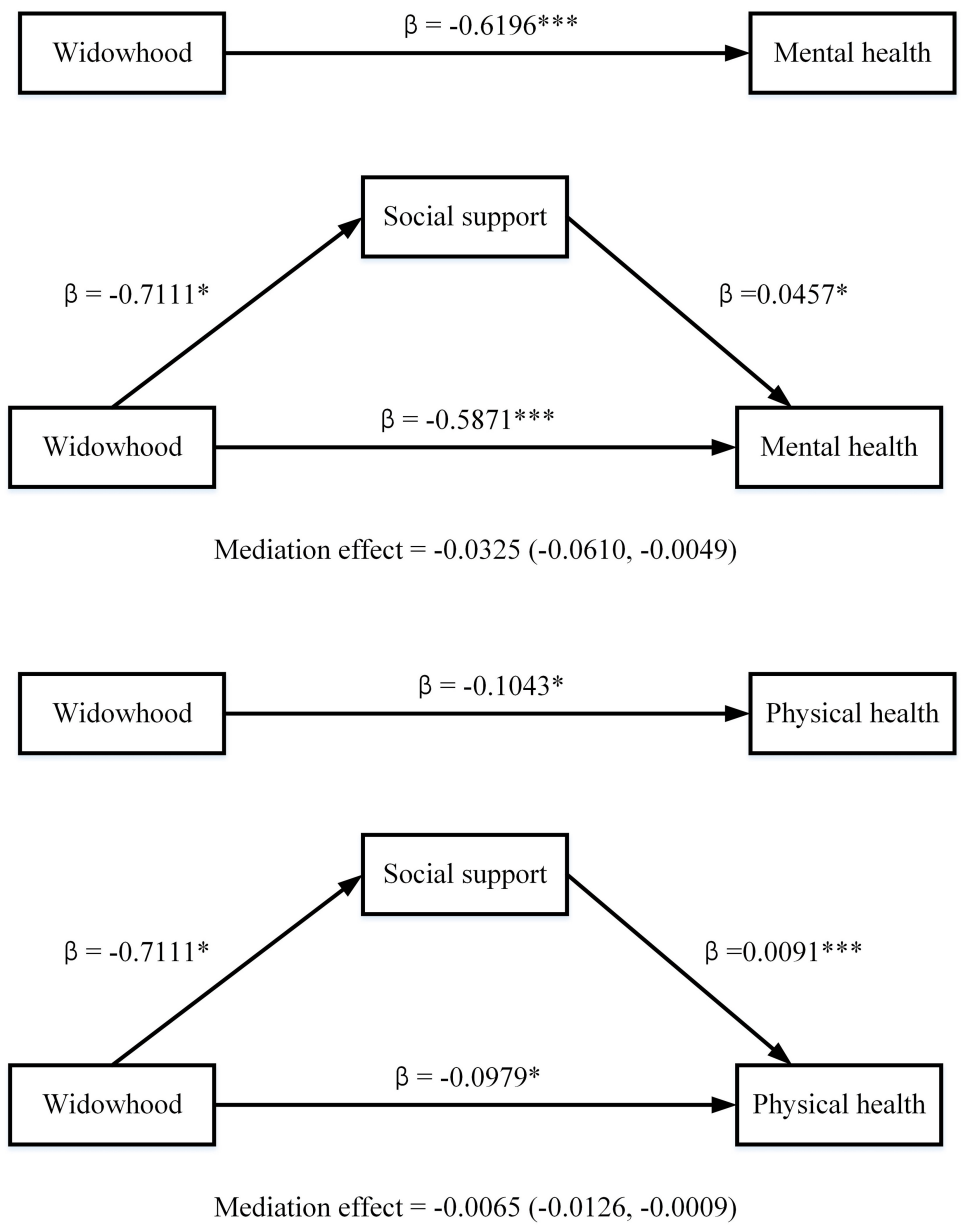
Mediating effects of social support in the association between widowhood and health (*N* = 7,647). Adjusting for gender, age, ethnicity, hukou, education, religion, social participation, and living arrangement; * *p* < 0.05, *** *p* < 0.001; Confidence intervals do not cross zero means the relationship is significant.

Figure 1 shows the results for mediation effects of social support between widowhood status and health status after controlling for socio-demographic characteristics. It can be seen that the coefficient of widowhood on both mental and physical health is reduced after the inclusion of social support. Widowhood has a significant and inverse relationship with social support, that is, older adults perceive less social support in widowhood (β = −0.7111, *p* < 0.05). Social support did exert a significant but small role in the relationship between widowhood and mental health [β = −0.0325, CI (−0.0610, −0.0049)] as well as physical health [β = −0.0065, CI (−0.0126, −0.0009)]. While it implies that widowhood reduces social support and further affects health.

Further analyses evaluate the subtypes of social support as mediators in the pathways from widowhood to health (see Table 2). The results revealed detective small mediation effects through emotional support and companionship for mental health [emotional support: β = −0.0076, CI (−0.0161, −0.0004); companionship: β = −0.0191, CI (−0.0338, −0.0063)]. For physical health, companionship was exerted as a significant mediator. However, the effects were also small [companionship: β = −0.009, CI (−0.0163, −0.0029)]. The direct effect of widowhood on mental and physical health was still significant, indicating that social support plays a partial mediating role. Partial mediation does not mean that the data results are not perfect, but implies that it is not the only relationship path.

**Table 2:** Mediation effects of the subtypes of social support in the association between widowhood and health status in the whole sample (*N* = 7,647).

**Health outcomes**	**Indirect effects**	**Coefficient**	**S.E**.	**Bootstrap 95%CI**	
				**Lower**	**Upper**
Mental health	Total	−0.0303	0.0139	−0.0572	−0.0030
	Emotional support	−0.0076	0.0041	−0.0161	−0.0004
	Instrumental support	−0.0036	0.0059	**−0.0153**	**0.0080**
	Companionship	−0.0191	0.0070	−0.0338	−0.0063
Physical health	Total	−0.0095	0.0034	−0.0165	−0.0031
	Emotional support	−0.0006	0.0012	**−0.0033**	**0.0018**
	Instrumental support	0.0001	0.0006	**−0.0012**	**0.0015**
	Companionship	−0.0090	0.0034	−0.0163	−0.0029

*S.E., standard errors; CI, confidence interval; Bold means not significant; Adjusted for gender, age, ethnicity, hukou, education, religion, social participation, and living arrangement*.

Then we conducted another set of analyses for different gender and rural-urban subsamples, reviewing the different role of social support in it. Significant mediation effects were only found in women and rural older adults. For women (see in Table 3), instrumental support and companionship have significant mediated effects in the relationship between widowhood and mental health [instrumental support: β = −0.0106, CI (−0.0243, −0.001); companionship: β = −0.0239, CI (−0.0419, −0.0087)]. Companionship significantly mediated the relationship between widowhood and physical health [companionship: β = −0.0172, CI (−0.0306, −0.0071)]. For males (see in Table 4), however, the mediating effect of social support was not significant because the impact of widowhood on social support was not significant.

**Table 3:** Mediation effects of the subtypes of social support in the association between widowhood and health status in the female subsample (*N* = 4,084).

**Health outcomes**	**Indirect effects**	**Coefficient**	**S.E**.	**Bootstrap 95%CI**	
				**Lower**	**Upper**
Mental health	Total	−0.0388	0.0118	−0.0625	−0.0155
	Emotional support	−0.0043	0.0035	**−0.0124**	**0.0010**
	Instrumental support	−0.0106	0.0059	−0.0243	−0.0010
	Companionship	−0.0239	0.0084	−0.0419	−0.0087
Physical health	Total	−0.0174	0.0057	−0.0302	−0.0076
	Emotional support	0.0021	0.0023	**−0.0013**	**0.0080**
	Instrumental support	−0.0023	0.0026	**−0.0084**	**0.0021**
	Companionship	−0.0172	0.0060	−0.0306	−0.0071

*If confidence intervals do not cross zero, then the relationship is significant; S.E., standard errors; CI, confidence interval; Bold means not significant; Adjusted for gender, age, ethnicity, hukou, education, religion, social participation, and living arrangement*.

**Table 4:** Mediation effects of the subtypes of social support in the association between widowhood and health status in the male subsample (*N* = 3,563).

**Health outcomes**	**Indirect effects**	**Coefficient**	**S.E**.	**Bootstrap 95%CI**	
				**Lower**	**Upper**
Mental health	Total	0.0015	0.0107	**−0.0193**	**0.0227**
	Emotional support	−0.0021	0.0027	**−0.0085**	**0.0022**
	Instrumental support	0.0058	0.0058	**−0.0047**	**0.0187**
	Companionship	−0.0022	0.0048	**−0.0122**	**0.0072**
Physical health	Total	−0.0076	0.0058	**−0.0194**	**0.0035**
	Emotional support	−0.0034	0.0030	**−0.0105**	**0.0011**
	Instrumental support	−0.0019	0.0025	**−0.0080**	**0.0018**
	Companionship	−0.0023	0.0049	**−0.0121**	**0.0072**

*If confidence intervals do not cross zero, then the relationship is significant; S.E., standard errors; CI, confidence interval; Bold means not significant; Adjusted for gender, age, ethnicity, hukou, education, religion, social participation, and living arrangement*.

For rural older adults (see in Table 5), the mediating effect of companionship in the pathways from widowhood to mental (β = −0.0175, CI (−0.0345, −0.0044)] and physical health was significant [physical heath: β = −0.0079, CI (−0.0173, −0.0012)]. For urban older adults (see in Table 6), the mediating effect of social support was not significant.

**Table 5:** Mediation effects of the subtypes of social support in the association between widowhood and health status in the rural subsample (*N* = 3,449).

**Health outcomes**	**Indirect effects**	**Coefficient**	**S.E**.	**Bootstrap 95%CI**	
				**Lower**	**Upper**
Mental health	Total	−0.0223	0.0150	**−0.0522**	**0.0071**
	Emotional support	−0.0039	0.0039	**−0.0131**	**0.0024**
	Instrumental support	−0.0010	0.0078	**−0.0169**	**0.0151**
	Companionship	−0.0175	0.0078	−0.0345	−0.0044
Physical health	Total	−0.0115	0.0053	−0.0225	−0.0017
	Emotional support	−0.0035	0.0031	**−0.0109**	**0.0011**
	Instrumental support	0.0000	0.0012	**−0.0027**	**0.0024**
	Companionship	−0.0079	0.0042	−0.0173	−0.0012

*If confidence intervals do not cross zero, then the relationship is significant; S.E., standard errors; CI, confidence interval; Bold means not significant; Adjusted for gender, age, ethnicity, hukou, education, religion, social participation, and living arrangement*.

**Table 6:** Mediation effects of the subtypes of social support in the association between widowhood and health status in the urban subsample (*N* = 4,198).

**Health outcomes**	**Indirect effects**	**Coefficient**	**S.E**.	**Bootstrap 95%CI**	
				**Lower**	**Upper**
Mental health	Total	−0.0094	0.0076	**−0.0247**	**0.0057**
	Emotional support	−0.0018	0.0022	**−0.0073**	**0.0016**
	Instrumental support	−0.0021	0.0034	**−0.0095**	**0.0042**
	Companionship	−0.0055	0.0043	**−0.0152**	**0.0018**
Physical health	Total	−0.0070	0.0052	**−0.0181**	**0.0029**
	Emotional support	0.0009	0.0016	**−0.0018**	**0.0048**
	Instrumental support	0.0004	0.0012	**−0.0018**	**0.0033**
	Companionship	−0.0083	0.0059	**−0.0205**	**0.0028**

*If confidence intervals do not cross zero, then the relationship is significant; S.E., standard errors; CI, confidence interval; Bold means not significant; Adjusted for gender, age, ethnicity, hukou, education, religion, social participation, and living arrangement*.

### Causal Mediation Analysis

Table 7 shows the results of causal mediation analysis. Becoming widowed was significantly associated with a mean score reduction in mental and physical health. When emotional support and companionship were modeled as mediators, the estimated indirect effect was significant, indicating the indirect effect through emotional support and companionship can explain the change in mental health and physical health. This is consistent with the findings of our main analysis.

**Table 7:** Causal mediation effects of the subtypes of social support in the association between widowhood and health status in the whole sample (*N* = 7,467).

**Health outcomes**	**Indirect effects**	**Coefficient**	**95%CI**	
			**Lower**	**Upper**
Mental health	Emotional support	−0.0285	−0.0517	−0.0078
	Instrumental support	**−0.0162**	−0.0415	0.0073
	Companionship	−0.0533	−0.0793	−0.0297
Physical health	Emotional support	−0.0051	**−0.0102**	**−0.0013**
	Instrumental support	−0.0028	**−0.0077**	**0.0013**
	Companionship	−0.0145	−0.0228	−0.0079

*If confidence intervals do not cross zero, then the relationship is significant; CI, confidence interval; Bold means not significant; Adjusted for gender, age, ethnicity, hukou, education, religion, social participation, and living arrangement*.

## Conclusion and Discussion

Using data from the 2014 wave of China Longitudinal Aging Social Survey, this study examined the mediation effects of social support, as well as different types including emotional support, instrumental support, and companionship, on the association between widowhood and older adults' health status. It was found that emotional support and companionship exerted a mediation effect on the influence of widowhood and health status. It indicates that older adults perceive significantly less emotional support and companionship after widowhood, leading to poorer mental health and physical health. Furthermore, women and rural older adults are more likely to be affected by the reduced emotional support and companionship caused by widowhood.

This study found that social support is an essential path through which widowhood is negatively associated with the health of old adults. It is consistent with other findings suggestive of the fact that social support explains part of the effects of widowhood on health (66, 79–81). Specifically, emotional support and companionship play a significant role in the relationship between widowhood and mental health as well as physical health. However, the contribution of social support here is small, as has also been found in other research exploring the mechanism of health outcomes (82–84). But this kind of small mediating role does exist, especially the mediating effect of emotional support and companionship.

Emotional support assumes a partial mediating role in the relationship between widowhood and mental health. It suggests that the negative effect of widowhood on the mental health of older adults is partly due to the reduced emotional support from family, relatives, and friends. This is consistent with previous studies which have shown that emotional support may be linked to widowhood and mental health (66, 85). And support is most helpful for widowed older adults to have the opportunity to freely express themselves (86). For older adults, a spouse is the main person to share private thoughts, boredom, depression. However, if a spouse who plays such an important role becomes deceased, it may create a void and, in the long run, may affect psychological health. And after the death of a spouse, older adults may be overwhelmed with grief and nostalgia for their spouse. For example, they will close themselves off and no longer share their hearts with others. This finding underscores the fact that emotional support is key in ameliorating the harmful effects of widowhood among older adults. Also, that older adults need communication and care to alleviate the intense psychological toll of widowhood.

Companionship plays an indispensable role in the pathway from widowhood to health outcomes. Companionship partially mediates the relationship between widowhood and health. That is, the negative association of widowhood with the mental and physical health of older adults is due to the decreased social interaction with family, relatives, and friends. Older people with close relationships tend to engage more in physical activities, have better dietary behavior, and have better access to information that may be beneficial to health (87, 88). However, the shrunken social network that is likely to characterize widowhood in older adults may be detrimental to health. Specifically, besides the void created when a spousal intimate relationship once relied upon is no longer available, widowed older adults may develop negative emotions like depression, pessimism, guilt, or fear of becoming a burden unto others. This may culminate in an unwillingness to interact with others or seek help. Such alienation amplifies the feelings of loneliness and loss, which may, in turn, impact physical functioning as well as both mental and physical health (89).

After taking gender into account, the mediation role of social support is only significant for women. This is consistent with some previous studies. Using data from Korea, Jeon found that social ties better explained the effect of widowhood on depression in women than in men (66). This phenomenon may be explained by the fact that with the transition to widowhood, women are more sensitive to such changes and tend to experience broader changes in social networks (90, 91). Furthermore, in a strongly patriarchal society such as China, women in marriage rely heavily on their husbands' status, power, and social resources, and are more affected by the changes caused by widowhood (92–94). In contrast, compared to women, elderly males have smaller social networks and are less likely to initiate or engage in social outdoor activities (52). Thus, widowhood has little effect on changes in social network size for elderly males, and the mediating role of social support is not significant either.

The mediating role of social support was only significant among rural older adults rather than urban older adults. That is, companionship significantly mediated the relationship between widowhood and mental as well as physical health in rural older adults. This may occur due to the disparities in the conception of marriage and the availability of social support between rural and urban areas (10, 95). In rural China, multigenerational cohabitation is very common, and social relationships are still dominated by family or clan; therefore, widowhood is more affected by changes in this network (56). While in urban areas, older adults have a wider social network and more social security resources, such as community-based care and scientific spiritual comfort. In addition, there are elderly colleges sponsored by the government and the community, to provide older residents with health information and creative enlightenment, as well as a place to meet new friends (96). As a result, the elderly have more opportunities to engage in social activities. All these may serve as a potential resource for social support, compensating for the decrease in social support resulting from the death of a spouse. Moreover, although it is seldom to hold multigenerational families in urban China, older adults tend to cohabitate with their children after being widowed, in order to provide care and nurturing for their grandchildren. This may provide a sense of belonging and continued engagement in family functions (97).

Our results did not find a significant difference in instrumental support between widowed and non-widowed older adults, and instrumental support did not play a mediating role in the relationship between widowhood and health status. Indeed, influenced by the Confucian ideology, the young generation should support the elderly, ensuring they are well-fed and clothed. Although this kind of support is mainly focused on material support and is always less mandatory in the aspects of spiritual, psychological care, and affection (98, 99), it does not change due to widowhood. So the mediating role of instrumental support is not significant.

This study still has several limitations to this study, which highlight the need for future research to better understand the connection between widowhood and health. First, analyses are limited by using self-report measures of mental and physical health scales, and they may differ when using objective assessments. Besides, we use the limitation of activities as a measure of physical health. While ADLs are an important component of health status, addressing functional status, other key components of health status in older adults include perceived health status, cognitive status, pain, and perceived quality of life, which need further research. Second, although we used a large sample size, it is cross-sectional data. This study only established a correlation but cannot make a causal inference. Further studies are needed to identify the causal mechanism of widowhood's effects on health based on longitudinal data. Thirdly, we only evaluated the partial mediation effects between widowhood and health status, and the effects of the mediator are small. Therefore, we urge some caution in interpreting our results. And there may likely be a case of missed variables that needs further research. For example, previous studies indicate that different sources and structures (relatives, friends, community, government agencies, etc.) of social support may differ in their functions (100–102). Thus, more work needs to be done to explore the role of social support in this association. Finally, it is also a limitation that CLASS doesn't provide other important contextual information about widowhood, such as time since the death of the spouse, age at the time of spouse's death, widowed person's perceived closeness to the spouse, or size of support system before widowhood. This limits our ability to meaningfully interpret the findings. Further research is needed.

Despite these limitations, our study extends the body of literature by providing evidence as to how widowhood may have deleterious effects on health, and how it differs across gender and urban vs. more remote residential areas. This study proposed that emotional support and companionship could potentially mediate the extent to which widowhood affects health. The evidence also suggests that it is only among rural and older women that social support plays a significant role in the association between widowhood and health. It underscores the evidence that emotional support and companionship may be associated with measures that improve the impact of widowhood on the health of older adults, particularly women and rural older adults.

## Data Availability Statement

Publicly available datasets were analyzed in this study. This data can be found here: http://class.ruc.edu.cn/.

## Ethics Statement

The studies involving human participants were reviewed and approved by the Ethics Committee of Renmin University of China. The patients/participants provided their written informed consent to participate in this study.

## Author Contributions

YG and TG designed the study, performed the statistical analysis, and drafted the manuscript. LM, LW, and JL collaborated with the study and revised the manuscript. All authors have given approval to the final version for publication.

## Funding

This work was supported by a Major Project of National Social Science Foundation of China (21ZDA103) and a National Social Science Foundation of China (18BRK012).

## Conflict of Interest

The authors declare that the research was conducted in the absence of any commercial or financial relationships that could be construed as a potential conflict of interest.

## Publisher's Note

All claims expressed in this article are solely those of the authors and do not necessarily represent those of their affiliated organizations, or those of the publisher, the editors and the reviewers. Any product that may be evaluated in this article, or claim that may be made by its manufacturer, is not guaranteed or endorsed by the publisher.

## Abbreviations

CLASS: The China Longitudinal Aging Social Survey
CES-D: Center for Epidemiological Studies Depression Scale
ADL: activities of daily living
S.E.: Standard errors
CI: confidence interval.
