# Supplementary material for: Widowhood and Health Status Among Chinese Older Adults: The Mediation Effects of Different Types of Support
Source: Front Public Health. 2021 Nov 17;9:745073. doi: 10.3389/fpubh.2021.745073 (PMC8637908; doi:10.3389/fpubh.2021.745073)
Supplement: Supplementary file 1 [file Table_1.pdf]

Supplementary Material

Table A1. Mediation effects of social support in the widowhood-health association by gender.

Health outcomes	Female (N=4084)			Male (N=3563)		
	Coefficient	Bootstrap 95%CI		Coefficient	Bootstrap 95%CI	
		Lower	Upper		Lower	Upper
Mental health	-0.0710	-0.1120	-0.0306	-0.0052	-0.0441	0.0352
Physical health	-0.0120	-0.0217	-0.0045	-0.0012	-0.0103	0.0073

Notes: If confidence intervals do not cross zero, then the relationship is significant; S.E.: standard errors; CI: confidence interval; Bold means not significant; Adjusted for gender, age, ethnicity, hukou, education, religion, social participation, and living arrangement.

Table A2. Mediation effects of social support in the widowhood-health association by rural/urban.

Health outcomes	Rural (N=3449)			Urban (N=4198)		
	Coefficient	Bootstrap 95%CI		Coefficient	Bootstrap 95%CI	
		Lower	Upper		Lower	Upper
Mental health	-0.0468	-0.0935	-0.0013	-0.0218	-0.0546	0.0097
Physical health	-0.0083	-0.0182	-0.0001	-0.0050	-0.0132	0.0023

Notes: If confidence intervals do not cross zero, then the relationship is significant; S.E.: standard errors; CI: confidence interval; Bold means not significant; Adjusted for gender, age, ethnicity, hukou, education, religion, social participation, and living arrangement.
